# Efficacy and safety of laparoscopic pancreaticoduodenectomy combined with a modified perioperative intraperitoneal chemotherapy regimen in resectable pancreatic head cancer: a dual-center retrospective cohort study

**DOI:** 10.3389/fonc.2025.1716199

**Published:** 2026-03-06

**Authors:** Ang Li, Yu Zhang, Yue Zhang, Jianhua Liu, Feng Feng, Chen Xu, Fengshan Li

**Affiliations:** 1 Department of Hepatobiliary and Pancreatic Surgery, The First Hospital of Hebei Medical University, Shijiazhuang, China; 2 Department of Hepatobiliary and Pancreatic Surgery, The Second Hospital of Hebei Medical University, Shijiazhuang, China

**Keywords:** laparoscopic pancreaticoduodenectomy, hyperthermic intraperitoneal chemotherapy, pancreatic cancer, overall survival, locoregional recurrence

## Abstract

**Background:**

Surgical resection for pancreatic cancer is associated with high rates of locoregional recurrence and peritoneal metastasis, leading to poor prognosis. This study aimed to evaluate the safety and efficacy of laparoscopic pancreaticoduodenectomy (LPD) combined with a modified perioperative hyperthermic intraperitoneal chemotherapy (HIPEC) regimen for resectable pancreatic head cancer.

**Methods:**

This dual-center retrospective cohort study included patients with resectable pancreatic head cancer who underwent LPD between May 2018 and July 2024. Patients were allocated to either the LPD-alone group (n=54) or the LPD plus HIPEC (LPD+HIPEC) group (n=55). The HIPEC protocol consisted of intraoperative hyperthermic saline perfusion, followed by intraperitoneal gemcitabine on postoperative day 2 and saline perfusion on day 4. The primary endpoint was overall survival (OS). Secondary endpoints included postoperative complications and patterns of recurrence. A multivariate Cox proportional hazards model was used to identify independent predictors of survival.

**Results:**

A total of 109 patients were analyzed. Baseline demographic, clinical, and key oncologic characteristics were comparable between the two groups. The incidence of major postoperative complications (Clavien-Dindo grade ≥III) was not significantly different between the LPD+HIPEC and LPD groups (5.5% vs. 5.6%, P = 1.000). The LPD+HIPEC group had a significantly longer median OS (27 months; 95% CI, 24.1–29.9) compared to the LPD group (23 months; 95% CI, 20.5–25.5; P = 0.045). The 1-, 2-, and 3-year OS rates were 84.9%, 58.2%, and 26.3% in the LPD+HIPEC group, versus 74.6%, 40.0%, and 15.0% in the LPD group, respectively. Locoregional recurrence was significantly lower in the LPD+HIPEC group (14.6% vs. 31.5%, P = 0.035). On multivariate analysis, treatment with LPD+HIPEC was an independent predictor of improved OS (Hazard Ratio: 0.58; 95% CI: 0.35–0.97; P = 0.038).

**Conclusion:**

In this retrospective analysis, LPD combined with a modified perioperative HIPEC regimen was associated with improved overall survival and reduced locoregional recurrence rates for resectable pancreatic head cancer, without a significant increase in severe postoperative morbidity. These findings suggest a potential therapeutic role for this strategy, warranting further investigation in prospective randomized trials.

## Introduction

Pancreatic cancer is a highly malignant neoplasm of the digestive system and ranks as the seventh leading cause of cancer-related mortality worldwide (1). For resectable pancreatic ductal adenocarcinoma (PDAC), radical surgical resection (R0 resection) remains the cornerstone of curative-intent treatment (2, 3). However, even after successful resection, long-term survival remains dismal, with 5-year survival rates for stage I-II disease at approximately 14%, and less than 5% for more advanced stages (4, 5). A primary driver of this poor outcome is the high rate of postoperative recurrence, which occurs in the majority of patients within two years. Common patterns of failure include distant metastases, primarily to the liver, and locoregional recurrence, including peritoneal seeding (6).

Peritoneal metastasis is a particularly challenging manifestation of recurrence, often leading to malignant ascites, bowel obstruction, and a significant decline in quality of life. The retroperitoneal location of the pancreas and the necessity of surgical manipulation can lead to the intraoperative shedding of tumor cells into the peritoneal cavity. Cytological studies have detected malignant cells in peritoneal lavage fluid in up to 33% of cases following resection, a significant increase from the 8% detection rate pre-resection (7). Once disseminated, these free-floating tumor cells are poorly vascularized, rendering systemic chemotherapy less effective (8).

Hyperthermic intraperitoneal chemotherapy (HIPEC) is a therapeutic modality designed to target and eradicate residual microscopic peritoneal disease. By delivering a heated cytotoxic solution directly into the abdominal cavity, HIPEC achieves high locoregional drug concentrations while minimizing systemic toxicity, with hyperthermia enhancing the cytotoxic effects of chemotherapy (9). While established in the treatment of other peritoneal surface malignancies, its role as an adjuvant to resection for primary pancreatic cancer is still emerging. Early studies, including a previous report from our group, have suggested that intraperitoneal hyperthermic perfusion may improve survival outcomes without imposing unacceptable risks (10). However, the optimal timing, drug selection, and technique for HIPEC in this context are not standardized, and robust evidence, particularly for its combination with minimally invasive laparoscopic pancreaticoduodenectomy (LPD), is limited.

This study was designed to address this knowledge gap by evaluating the safety and efficacy of LPD combined with a modified, multi-stage perioperative HIPEC protocol at two high-volume centers. We hypothesized that this combined approach could reduce locoregional recurrence and improve survival outcomes compared to LPD alone, without increasing severe postoperative complications.

## Materials and methods

### Study design and patient population

This dual-center retrospective cohort study was conducted in accordance with the Strengthening the Reporting of Observational Studies in Epidemiology (STROBE) guidelines (11). We analyzed data from consecutive patients with resectable pancreatic head cancer who underwent LPD at the First and Second Hospitals of Hebei Medical University between May 2018 and July 2024. The study was approved by the Ethics Committee of the First Hospital of Hebei Medical University, and written informed consent was obtained from all participants.

Inclusion criteria were: (1) age ≥18 years; (2) pathologically confirmed pancreatic ductal adenocarcinoma of the head; (3) classification of resectable disease based on National Comprehensive Cancer Network (NCCN) criteria (12); (4) underwent LPD with curative intent; and (5) able to receive the standard postoperative adjuvant chemotherapy regimen (gemcitabine plus capecitabine). Exclusion criteria were: (1) receipt of neoadjuvant chemotherapy or radiotherapy; (2) intraoperative discovery of distant metastases; (3) requirement for vascular or other extra-pancreatic organ resection; (4) presence of other synchronous malignancies; (5) contraindications to HIPEC or general anesthesia; and (6) postoperative inability or refusal to complete adjuvant chemotherapy.

**Figure 1:**
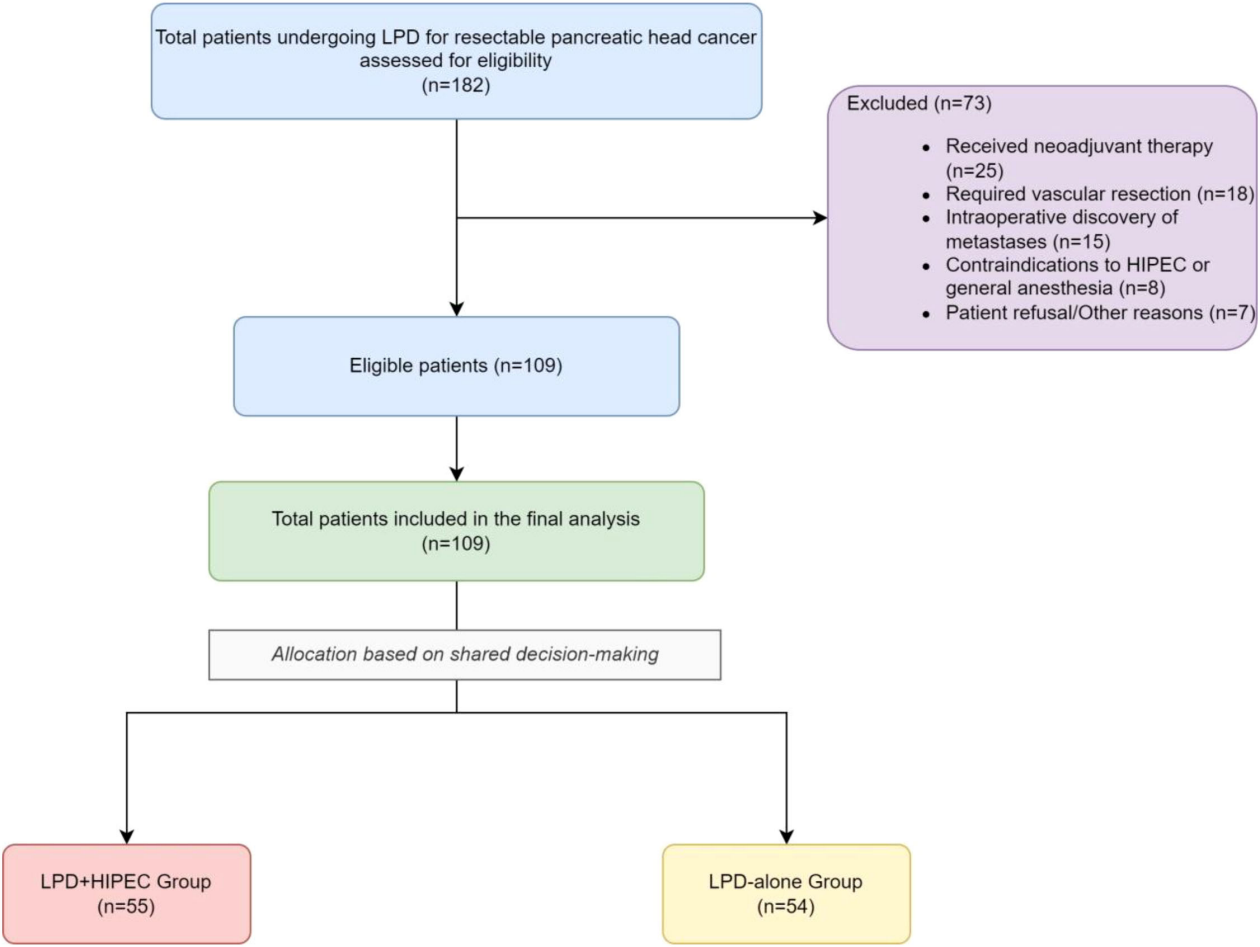
STROBE flow diagram of patient selection. This diagram illustrates the screening of patients, reasons for exclusion, and the final allocation of patients to the two study cohorts.

Starting in 2018, our institutions introduced the modified HIPEC protocol as a treatment option. All eligible patients who met the study’s inclusion criteria were presented with both treatment options (LPD alone or LPD with HIPEC) during preoperative counseling. The discussion included the established nature of LPD, the investigational status of the HIPEC addition, its theoretical benefits in reducing local recurrence, and the potential for increased systemic inflammation and unknown risks. The final treatment allocation was based on the patient’s voluntary and informed decision after this shared decision-making process, without a pre-defined clinical protocol for patient selection into either arm. A total of 109 patients who met the criteria were enrolled and divided into two groups: the LPD-alone group (n=54) and the LPD plus HIPEC (LPD+HIPEC) group (n=55). A total of 109 patients who met the criteria were enrolled and divided into two groups: the LPD-alone group (n=54) and the LPD plus HIPEC (LPD+HIPEC) group (n=55), as detailed in the patient flow diagram (**Figure 1**).

### Treatment procedures

#### Laparoscopic pancreaticoduodenectomy

All LPD procedures were performed by experienced surgeons with equivalent qualifications and technical proficiency. The surgical technique was standardized. After laparoscopic exploration to exclude occult metastases, a standard LPD was performed. This involved Kocherization of the duodenum, dissection and transection of the gastric antrum and proximal jejunum, and division of the pancreatic neck. The specimen, encompassing the pancreatic head, duodenum, gallbladder, distal bile duct, and regional lymph nodes, was fully mobilized and removed. Gastrointestinal continuity was restored via pancreaticojejunostomy, choledochojejunostomy, and gastrojejunostomy. For the pancreaticojejunostomy, a variable-diameter pancreatic duct catheter was utilized as previously described by our team to minimize leakage (13).

#### Modified perioperative HIPEC protocol

For patients in the LPD+HIPEC group, a multi-stage protocol was implemented.

**Figure 2:**
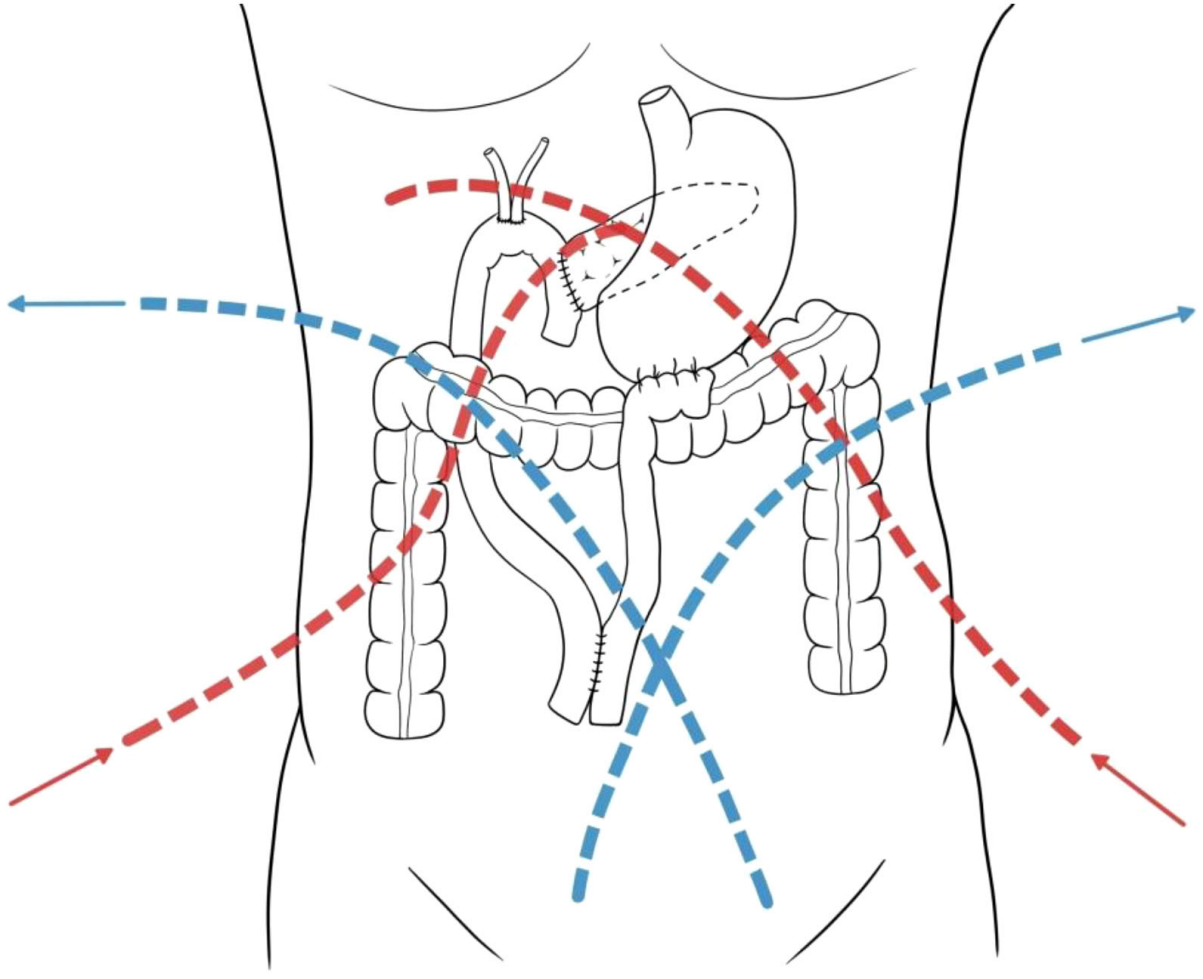
Schematic of catheter placement for HIPEC. The red dashed lines indicate the paths of the inflow tubes, placed anterior to the pancreaticojejunal anastomosis and posterior to the bilioenteric anastomosis, respectively. The blue dashed line indicates the path of the outflow tubes placed crosswise in the pelvis.

Stage 1 (Intraoperative): Following the completion of all anastomoses and prior to abdominal closure, two inflow catheters were placed (one anterior to the pancreaticojejunostomy, one posterior to the choledochojejunostomy) and two outflow catheters were placed crosswise in the pelvis (**Figure 2**). A 40-minute perfusion of heated normal saline (2000 mL/m^2^) was performed. The inflow temperature was maintained at 43.0 ± 0.2°C and the outflow temperature at 40.0 ± 0.2 °C, with a perfusion rate of 450–600 mL/min. The patient’s abdomen was manually agitated to ensure uniform distribution.

Stage 2 (Postoperative day 2): A second perfusion was administered in the ward using gemcitabine (1000 mg/m^2^) diluted in 2000 mL/m^2^ of normal saline. The temperature and duration parameters were identical to the intraoperative session.

Stage 3 (Postoperative day 4): A final 40-minute perfusion with heated normal saline (2000 mL/m^2^) was performed to lavage the abdominal cavity.

Patient safety during perfusion was ensured by continuous monitoring of core body temperature, electrocardiogram, and hemodynamics. Intraperitoneal temperature was verified using thermistor probes on both the inflow and outflow lines to ensure the target range was maintained.

#### Postoperative management and adjuvant chemotherapy

All patients received standardized postoperative care. Within 8 weeks of surgery, patients in both groups commenced a systemic adjuvant chemotherapy regimen consisting of gemcitabine and capecitabine, as per institutional protocol.

### Data collection and follow-up

We collected data on patient demographics, preoperative clinical status, intraoperative variables, and postoperative outcomes. Postoperative complications were recorded and graded according to the Clavien-Dindo classification (14). Postoperative pancreatic fistula (POPF) was defined and graded by the International Study Group on Pancreatic Surgery (ISGPS) 2016 criteria (15).

Patients were followed up every 3 months for the first 2 years and every 6 months thereafter, until July 2024 or death. Follow-up assessments included physical examination, serum tumor marker (CA19-9) measurement, and contrast-enhanced computed tomography (CT) of the chest and abdomen. Follow-up was conducted according to a standardized institutional protocol for all postoperative pancreatic cancer patients, irrespective of their treatment group. This protocol included contrast-enhanced CT scans of the chest and abdomen every 3–4 months for the first two years, and every 6 months thereafter. All radiological scans were independently reviewed by two senior radiologists blinded to the treatment allocation. In cases of equivocal findings, follow-up imaging within 4–6 weeks or a PET-CT scan was recommended to confirm or rule out recurrence. Recurrence was defined by radiological evidence of new lesions on contrast-enhanced CT, with biopsy confirmation when clinically feasible. Locoregional recurrence was defined as disease relapse at the surgical resection bed, anastomotic sites, peritoneal surfaces (visceral or parietal peritoneum, omentum, and mesentery), or in regional lymph node basins (including peripancreatic, superior mesenteric, and para-aortic nodes corresponding to AJCC 8th edition nodal stations). Overall survival (OS) was calculated from the date of surgery to the date of death from any cause.

### Statistical analysis

All statistical analyses were performed using SPSS software version 26.0 (IBM Corp., Armonk, NY, USA). Continuous variables were expressed as mean ± standard deviation (SD) or median (interquartile range, IQR) and compared using the Student’s t-test or Mann-Whitney U test, as appropriate. Categorical variables were expressed as numbers (percentages) and compared using the Chi-square test or Fisher’s exact test. Standardized mean differences (SMDs) were calculated to assess the balance of baseline characteristics, with an SMD < 0.2 considered to indicate good balance. Survival curves were generated using the Kaplan-Meier method and compared with the log-rank test. A multivariate Cox proportional hazards regression analysis was performed to identify independent predictors of OS. The selection of covariates for the multivariable model was based on a combination of clinical relevance and statistical significance in univariate analysis. Specifically, variables with a P-value < 0.10 on univariate analysis were considered for inclusion, along with the primary treatment variable (LPD+HIPEC vs. LPD), to construct a parsimonious model that adjusts for key potential confounders while minimizing the risk of overfitting. The proportional hazards assumption for the Cox model was assessed by examining the Schoenfeld residuals, and no significant violations were detected for any of the included covariates (P > 0.05 for all). Hazard ratios (HR) and 95% confidence intervals (CI) were calculated. A two-sided P-value < 0.05 was considered statistically significant. There were no missing data for the primary or secondary endpoints among the 109 patients included in the final analysis.

## Results

### Patient characteristics

A total of 109 patients met the inclusion criteria, with 55 in the LPD+HIPEC group and 54 in the LPD-alone group. The baseline demographic and clinical characteristics were well-balanced between the two cohorts, with no significant differences in age, sex, BMI, comorbidities, or preoperative CA19–9 levels (**Table 1**). Crucially, key prognostic oncologic and surgical factors, including preoperative tumor size, ECOG performance status, pathological T/N stage, and resection margin status, were also comparable between the groups (**Table 2**). The excellent balance was further confirmed by the standardized mean differences, all of which were below 0.2.

**Table 1:** Comparison of demographic and clinical characteristics between the groups.

Characteristic	LPD+HIPEC (n=55)	LPD (n=54)	Test Value	P-value	SMD
Age (years), mean ± SD	61.82 ± 6.90	60.89 ± 9.07	t=-0.603	0.548	0.116
Sex, n (%)			χ^2^ = 1.194	0.275	0.207
Male	30 (54.5)	35 (64.8)			
Female	25 (45.5)	19 (35.2)			
BMI (kg/m^2^), mean ± SD	24.26 ± 2.72	23.42 ± 3.27	t=-1.464	0.146	0.279
Serum Albumin (g/L), mean ± SD	39.02 ± 3.50	38.57 ± 4.33	t=-0.594	0.554	0.113
Preoperative biliary drainage, n (%)	20 (36.4)	16 (29.6)	χ^2^ = 0.559	0.455	0.141
Comorbidities, n (%)					
Hypertension	17 (30.9)	21 (38.9)	χ^2^ = 0.764	0.382	0.169
Diabetes	4 (7.3)	8 (14.8)	χ^2^ = 1.582	0.208	0.245
Coronary heart disease	1 (1.8)	4 (7.4)	*	0.206	0.264
Preoperative CA19-9 (U/mL), median (IQR)	60.7 (25.1, 150.3)	44.7 (20.5, 135.8)	Z=-1.212	0.225	0.188

LPD, Laparoscopic Pancreaticoduodenectomy; HIPEC, Hyperthermic Intraperitoneal Chemotherapy; SD, Standard Deviation; BMI, Body Mass Index; IQR, Interquartile Range; SMD, Standardized Mean Difference. An SMD < 0.2 was considered to indicate a good balance between the two groups.

*Fisher’s exact test.

**Table 2:** Comparison of oncologic and pathological characteristics.

Characteristic	LPD+HIPEC (n=55)	LPD (n=54)	Test Value	P-value	SMD
ECOG Performance Status, n (%)			χ^2^ = 0.231	0.631	0.052
0	40 (72.7)	38 (70.4)			
1	15 (27.3)	16 (29.6)			
Tumor Size on pathology (cm), mean ± SD	2.8 ± 0.9	3.0 ± 1.1	t=0.985	0.327	0.198
Pathological AJCC 8^th^ T stage, n (%)			χ^2^ = 0.875	0.646	0.098
T1	11 (20.0)	10 (18.5)			
T2	29 (52.7)	31 (57.4)			
T3	15 (27.3)	13 (24.1)			
Pathological AJCC 8^th^ N stage, n (%)			χ^2^ = 0.452	0.798	0.195
N0	23 (41.8)	27 (50.0)			
N1	28 (50.9)	20 (37.0)			
N2	4 (7.3)	7 (13.0)			
Resection Margin, n (%)			*	0.751	0.059
R0 (negative)	47 (85.5)	45 (83.3)			
R1 (positive)	8 (14.5)	9 (16.7)			
Lymphovascular invasion, n (%)	21 (38.2)	24 (44.4)	χ^2^ = 0.432	0.511	0.126
Perineural invasion, n (%)	35 (63.6)	32 (59.3)	χ^2^ = 0.229	0.632	0.089

ECOG, Eastern Cooperative Oncology Group; AJCC, American Joint Committee on Cancer; SMD, Standardized Mean Difference. An SMD < 0.2 was considered to indicate a good balance between the two groups.

*Fisher’s exact test.

### Intraoperative and postoperative outcomes

All 109 LPD procedures were completed successfully without conversion to open surgery. The intraoperative and postoperative outcomes are detailed in **Table 3**. There was no significant difference in mean surgical time or median intraoperative blood loss between the groups. A detailed breakdown of postoperative complications according to the Clavien-Dindo classification is provided in **Table 4**. The overall incidence of postoperative complications was similar. Notably, there was no statistically significant difference in the rate of clinically relevant POPF (Grade B/C) (5.5% in LPD+HIPEC vs. 9.3% in LPD; P = 0.489). The incidence of severe complications (Clavien-Dindo grade ≥III) was also comparable (5.5% vs. 5.6%; P = 1.000). The two in-hospital mortalities in the LPD+HIPEC group involved a 72-year-old male with pre-existing diabetes and hypertension who succumbed to sepsis from a Grade C pancreatic fistula, and a 68-year-old male with coronary artery disease who suffered a fatal postoperative hemorrhage. Both patients had a borderline preoperative performance status (ECOG 1) and mild hypoalbuminemia. No in-hospital deaths occurred in the LPD-alone group. Patients in the LPD+HIPEC group had a significantly higher maximum body temperature on postoperative day 5 (38.28 ± 0.70 °C vs. 37.77 ± 0.51 °C; P<0.001), consistent with the systemic inflammatory response to hyperthermic perfusion. Systemic inflammatory markers were transiently elevated in the LPD+HIPEC group, with significantly higher peak C-reactive protein levels (185 ± 42 mg/L vs. 132 ± 35 mg/L, P<0.001) and white blood cell counts (14.5 ± 3.1 x10^9^/L vs. 11.8 ± 2.5 x10^9^/L, P<0.001) compared to the LPD group. These values typically normalized within 5–7 days. Other transient HIPEC-related adverse events were minimal (**Supplementary Table S1**).

**Table 3:** Comparison of intraoperative and general postoperative outcomes.

Variable	LPD+HIPEC (n=55)	LPD (n=54)	Test Value	P-value
Surgical time (min), mean ± SD	319.85 ± 56.20	326.78 ± 51.13	t=0.672	0.503
Intraoperative blood loss (mL), median (IQR)	300 (200, 500)	300 (200, 400)	Z=-0.588	0.556
Max temperature on POD 5 (°C), mean ± SD	38.28 ± 0.70	37.77 ± 0.51	t=-4.290	<0.001
Postoperative hospital stay (days), median (IQR)	14 (11, 19)	13 (10, 16)	Z=-1.852	0.064

POD, Postoperative Day.

**Table 4:** Detailed postoperative complications according to the Clavien-Dindo classification.

Complication	LPD+HIPEC (n=55), n (%)	LPD (n=54), n (%)	P-value
Grade I			
Postoperative Ileus (self-resolved)	5 (9.1)	4 (7.4)	0.748
Grade II (requiring medication)			
Urinary Tract Infection	3 (5.5)	2 (3.7)	0.679
Superficial Wound Infection	2 (3.6)	1 (1.9)	0.615
Grade IIIa (requiring intervention without general anesthesia)			
Percutaneous drainage for collection	1 (1.8)	1 (1.9)	1.000
Grade IIIb (requiring intervention with general anesthesia)			
Reoperation for bleeding	1 (1.8)	1 (1.9)	1.000
Reoperation for anastomotic leak	0 (0)	1 (1.9)	0.495
Grade Iva (single organ dysfunction)	0 (0)	1 (1.9)	0.495
Grade V (In-hospital mortality)	**2 (3.6)**	**0 (0)**	**0.242**
Total patients with complications (any grade)	**13 (23.6)**	**11 (20.4)**	**0.676**
Severe complications (Grade ≥III)	**5 (9.1)**	**4 (7.4)**	**0.748**

Bold values indicate statistical significance (P < 0.05).

### Adjuvant therapy and follow-up

Details of adjuvant chemotherapy administration are presented in **Table 5**. The proportion of patients who initiated and completed the planned adjuvant therapy regimen was similar between the LPD+HIPEC and LPD groups (85.5% vs. 83.3%, P = 0.748), suggesting that the HIPEC procedure did not impair tolerance to subsequent systemic treatment. The median follow-up duration for the entire cohort was 15 months (range, 1–36 months). The median follow-up was longer in the LPD+HIPEC group (18 months) compared to the LPD group (12 months).

**Table 5:** Comparison of postoperative adjuvant chemotherapy.

Variable	LPD+HIPEC (n=55)	LPD (n=54)	Test value	P-value
Time to adjuvant therapy (weeks), mean ± SD	6.1 ± 1.2	5.9 ± 1.3	t=-0.831	0.408
Patients commencing adjuvant therapy, n (%)	51 (92.7)	50 (92.6)	*	1.000
Patients completing planned therapy, n (%)	47 (85.5)	45 (83.3)	χ^2^ = 0.103	0.748
Reason for discontinuation, n				
Toxicity/Intolerance	3	4		
Patient refusal/Other	1	1		

*Fisher’s exact test.

### Recurrence and survival outcomes

Recurrence patterns are summarized in **Table 6**. At the final follow-up, the LPD+HIPEC group exhibited a significantly lower rate of locoregional recurrence compared to the LPD group (14.6% vs. 31.5%, P = 0.035). Within the locoregional recurrence category, nodal recurrence was observed in 9 patients (16.7%) in the LPD group and 4 patients (7.3%) in the LPD+HIPEC group (**Table 6**). There were no significant differences in the rates of liver or lung metastases between the two cohorts.

**Table 6:** Comparison of postoperative follow-up and recurrence patterns.

Characteristic	LPD+HIPEC (n=55)	LPD (n=54)	Test value	P-value
Postoperative CA19-9 (U/mL), median (IQR)	29.16 (11.62, 44.53)	29.54 (13.71, 39.72)	Z=-1.112	0.911
Recurrence Patterns, n (%)				
Locoregional recurrence (overall)	8 (14.6)	17 (31.5)	χ^2^ = 4.422	0.035
Nodal Recurrence (as part of locoregional)	4 (7.3)	9 (16.7)	*	0.141
Liver metastasis	13 (23.6)	10 (18.5)	χ^2^ = 0.429	0.513
Lung metastasis	6 (10.9)	4 (7.4)	χ^2^=0.401	0.527

CA19-9 measured at 3 months postoperatively.

*Fisher’s exact test.

**Figure 3:**
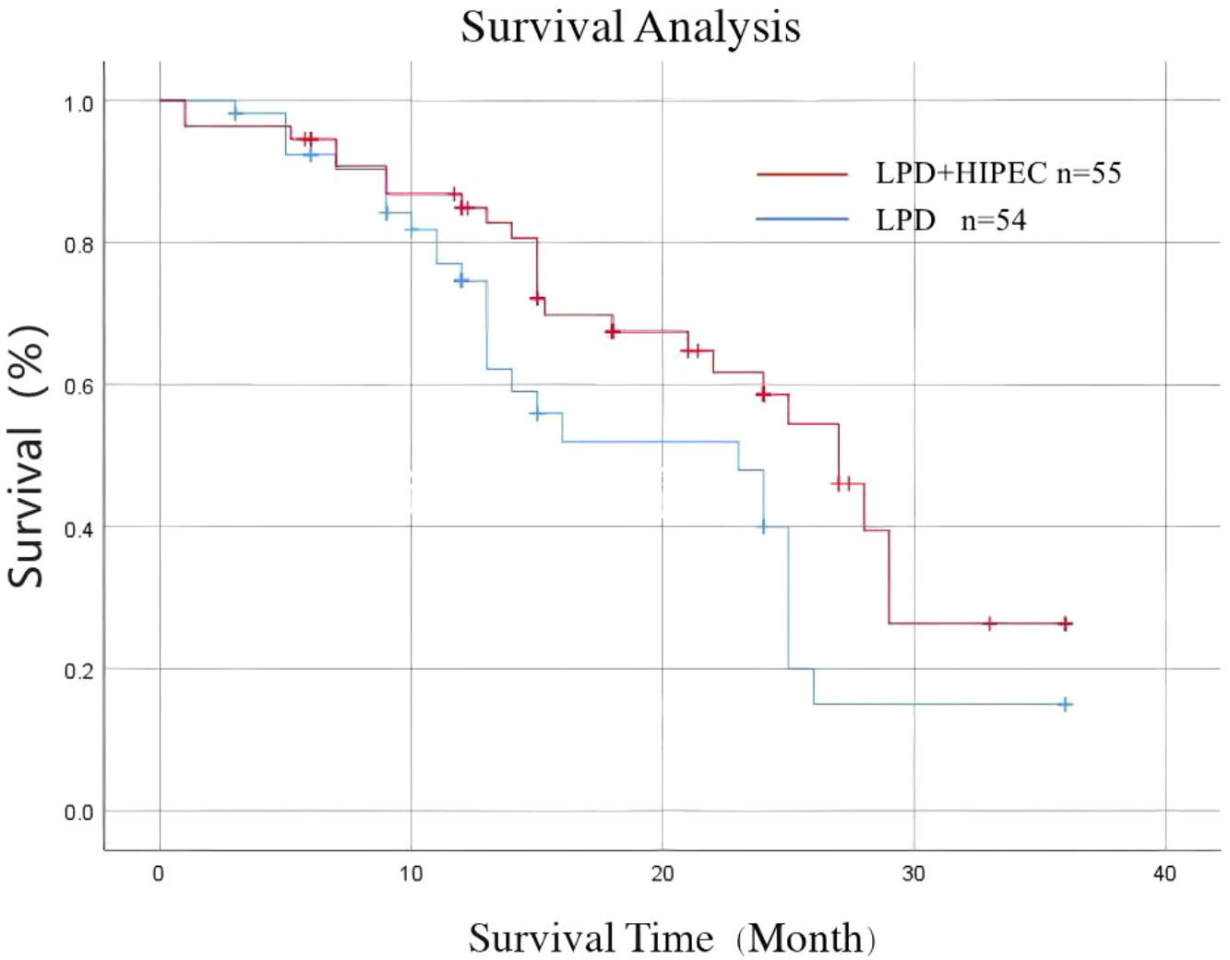
Kaplan-Meier curves for overall survival. Comparison of overall survival between the LPD+HIPEC group (n=55) and the LPD-alone group (n=54). The median overall survival was 27 months in the LPD+HIPEC group and 23 months in the LPD group (Log-rank test, P = 0.045).

The Kaplan-Meier survival analysis demonstrated a significant benefit for the LPD+HIPEC group (**Figure 3**). The median OS was 27 months (95% CI, 24.1–29.9 months) in the LPD+HIPEC group, compared to 23 months (95% CI, 20.5–25.5 months) in the LPD group (log-rank P = 0.045). The 1-year, 2-year, and 3-year OS rates in the LPD+HIPEC group were 84.9%, 58.2%, and 26.3%, respectively. In the LPD group, the corresponding rates were 74.6%, 40.0%, and 15.0%.

On multivariate Cox regression analysis, treatment with LPD+HIPEC (HR 0.58, 95% CI 0.35–0.97, P = 0.038), pathological N0 stage (HR 0.45, 95% CI 0.26–0.78, P = 0.004), and R0 resection (HR 0.51, 95% CI 0.27–0.95, P = 0.034) were identified as independent prognostic factors for improved overall survival (**Table 7**).

**Table 7:** Multivariate Cox regression analysis of predictors for overall survival.

Variable	Hazard Ratio (HR)	95% Confidence Interval (CI)	P-value
Treatment Group (LPD+HIPEC vs. LPD)	**0.58**	**0.35 - 0.97**	**0.038**
Age (≥65 vs. <65 years)	1.21	0.72 - 2.03	0.471
Pathological T stage (T3 vs. T1/T2)	1.55	0.91 - 2.64	0.105
Pathological N stage (N+ vs. N0)	**2.21**	**1.30 - 3.76**	**0.004**
Resection Margin (R1 vs. R0)	**1.92**	**1.03 - 3.58**	**0.034**
Lymphovascular invasion (Present vs. Absent)	1.48	0.88 - 2.49	0.138

Bold values indicate statistical significance (P < 0.05).

## Discussion

In this dual-center retrospective study, we investigated the impact of adding a modified perioperative HIPEC regimen to standard LPD for resectable pancreatic head cancer. Our principal finding is that this combination therapy was associated with a significant improvement in overall survival and a reduction in locoregional recurrence compared to LPD alone. Importantly, these oncologic benefits did not come at the cost of a significant increase in severe postoperative morbidity.

The rationale for employing intraperitoneal chemotherapy in pancreatic cancer is compelling. The high propensity for peritoneal seeding after curative-intent resection necessitates an effective locoregional therapy. Our results align with this concept, demonstrating a more than two-fold reduction in locoregional recurrence in the HIPEC group. This finding suggests that the perioperative delivery of hyperthermic chemotherapy can effectively eradicate the microscopic residual tumor cells that are shed during surgery, thereby delaying or preventing peritoneal failure. This is consistent with previous studies that have also shown a positive effect of intraperitoneal chemotherapy on controlling local disease (10, 16).

A key aspect of our study is the novel, multi-stage HIPEC protocol. Conventional HIPEC involves a single intraoperative administration of chemotherapy. Our protocol included an initial intraoperative hyperthermic lavage with saline, followed by delayed gemcitabine administration on postoperative day 2. This approach is conceptually a hybrid between classic HIPEC and early postoperative intraperitoneal chemotherapy (EPIC). The initial intraoperative hyperthermic perfusion serves to mechanically lavage the peritoneal cavity while the hyperthermia itself may induce apoptosis in cancer cells and enhance drug penetration (17, 18). Delaying the cytotoxic agent to POD 2 allows for patient hemodynamic stabilization after major surgery, potentially increasing safety, a concept supported by early EPIC studies for other malignancies (19, 20). This timing still falls within the critical window before the formation of significant postoperative adhesions, which could otherwise limit the uniform distribution and efficacy of the intraperitoneal chemotherapy. Gemcitabine was chosen due to its known efficacy in pancreatic cancer and preclinical evidence demonstrating a synergistic cytotoxic effect when combined with hyperthermia (21, 22).

The safety of combining HIPEC with a complex procedure like LPD, particularly with respect to anastomotic integrity, is a major concern. Postoperative pancreatic fistula remains a dreaded complication. In our cohort, the rate of clinically relevant POPF was not significantly increased in the LPD+HIPEC group, nor were the rates of other major complications. This finding suggests that our modified protocol is feasible and relatively safe in a high-volume setting. The extensive surgical experience of our centers, having performed over 2,000 LPDs since 2013, likely contributed to the low overall complication rates. However, the two in-hospital deaths in the HIPEC group, although not statistically significant in this sample size, are a sobering reminder of the potential toxicity and must be considered when evaluating the risk-benefit profile. This underscores the need for careful patient selection for such an aggressive combined-modality treatment.

Our findings are consistent with some earlier studies of HIPEC in the open surgery setting. For instance, Tentes et al. reported promising survival benefits with intraoperative HIPEC after pancreatoduodenectomy (16). However, our study is one of the few to evaluate this approach in a purely laparoscopic context and with a delayed, multi-stage chemotherapy regimen, which may offer a different safety and efficacy profile. The primary outcome of our study, a 4-month improvement in median OS, which remained significant on multivariate analysis, is clinically meaningful in the context of pancreatic cancer. While this survival benefit is encouraging, it must be interpreted with caution given the study’s limitations.

The statistical significance of the primary endpoint, overall survival, was marginal (P = 0.045), which should be interpreted with caution. This is likely attributable to the limited statistical power of our study due to its modest sample size. A larger cohort might have demonstrated a more robust statistical difference. However, the observed absolute improvement of 4 months in median OS, coupled with a hazard ratio of 0.58 in the multivariable analysis, represents a clinically meaningful effect size in the context of this aggressive disease, where incremental survival gains are hard-won (23). This survival benefit is further corroborated by the substantial and statistically significant reduction in locoregional recurrence, which is the primary mechanistic endpoint for a locoregional therapy like HIPEC (24). Therefore, while statistically borderline, the convergence of evidence from survival analysis and recurrence patterns suggests a genuine therapeutic effect that warrants further investigation.

### Limitations

This study has several important limitations. First and foremost, its retrospective, non-randomized design is susceptible to selection bias. Although we demonstrated that the groups were well-matched across a wide range of demographic and key oncologic variables, and our multivariate analysis adjusted for key confounders, unmeasured biases may still exist. The decision to offer HIPEC was based on a shared decision-making process, which could have inadvertently selected for patients perceived to be fitter candidates. A sensitivity analysis using methods such as propensity score matching (PSM) was considered to further mitigate potential selection bias. However, given the modest sample size of our cohort, implementing PSM would have significantly reduced the statistical power and potentially introduced instability in the estimates. Therefore, we opted for multivariable Cox regression as the primary method to adjust for confounders, acknowledging this as a limitation. Second, the sample size is relatively small, which may limit the statistical power to detect smaller differences in complication rates and strengthens the need for cautious interpretation of marginally significant p-values. Third, there was a discrepancy in the median follow-up duration between the two groups, with a shorter follow-up in the control arm. This could potentially underestimate late recurrences in the LPD-alone group, though the statistically significant separation of the Kaplan-Meier curves early on suggests a true treatment effect. Additionally, the reliance on radiological rather than universal biopsy-proven recurrence could introduce measurement bias. Finally, this is a report of an institutional experience with a non-standardized HIPEC protocol, and the results may not be generalizable to centers with different protocols or lower surgical volumes.

### Future directions

Based on our findings, we hypothesize that optimal candidates for this intensive regimen are patients aged <70 years with excellent performance status (ECOG 0), minimal comorbidities, and robust nutritional status. Furthermore, patients with tumors exhibiting high-risk features for peritoneal dissemination, such as T3 stage or positive peritoneal cytology, may derive the greatest benefit. Our future plans include a prospective Phase II trial to validate the safety and efficacy of this protocol in a more stringently selected population, incorporating biomarkers such as circulating tumor DNA (ctDNA) to stratify risk and monitor therapeutic response.

## Conclusion

In conclusion, this dual-center retrospective cohort study suggests that LPD combined with a modified perioperative HIPEC regimen is associated with improved overall survival and decreased locoregional recurrence in patients with resectable pancreatic head cancer, with an acceptable safety profile in a high-volume setting. While these findings are promising, they should be considered hypothesis-generating. Rigorously designed, multi-center randomized controlled trials are imperative to definitively establish the role of this combined-modality approach in the standard of care for pancreatic cancer.

## Data Availability

The original contributions presented in the study are included in the article/**Supplementary Material**. Further inquiries can be directed to the corresponding author.

## Supplementary material

Supplementary Table 1 Comparison of postoperative systemic inflammatory response and transient HIPEC-related adverse events.
