# Supplementary material for: Efficacy and safety of laparoscopic pancreaticoduodenectomy combined with a modified perioperative intraperitoneal chemotherapy regimen in resectable pancreatic head cancer: a dual-center retrospective cohort study
Source: Front Oncol. 2026 Mar 6;15:1716199. doi: 10.3389/fonc.2025.1716199 (PMC13002443; doi:10.3389/fonc.2025.1716199)
Supplement: Supplementary Table 1 — Comparison of postoperative systemic inflammatory response and transient HIPEC-related adverse events. [file Table1.docx]

**Supplementary Table S1. Comparison of postoperative systemic inflammatory response and transient HIPEC-related adverse events.**

| **Variable** | **LPD+HIPEC (n=55)** | **LPD (n=54)** | **P-value** |
| --- | --- | --- | --- |
| **Peak C-reactive protein (mg/L), mean ± SD** | 185 ± 42 | 132 ± 35 | <0.001 |
| **Peak white blood cell count (x10^9^/L), mean ± SD** | 14.5 ± 3.1 | 11.8 ± 2.5 | <0.001 |
| **Transient hypotension during perfusion, n (%)** | 3 (5.5) | N/A | - |
| **Grade 1 acute kidney injury (transient), n (%)** | 4 (7.3) | 1 (1.9) | 0.171 |
| **Nausea/vomiting (Grade 1/2), n (%)** | 12 (21.8) | 7 (13.0) | 0.198 |

Data are presented as n (%) or mean ± SD. Hypotension defined as requiring vasopressor support during perfusion. N/A, not applicable.
